# Perception of maxillary incisor inclination and its correlation with dental cephalometric measurements

**DOI:** 10.1177/14653125241248663

**Published:** 2024-04-29

**Authors:** Sara Palmares, Rui Caseiro, Rui Pereira, Luís Jardim

**Affiliations:** 1 Department of Orthodontics, School of Dentistry, University of Lisbon, Lisbon, Portugal; 2 Orthodontics’ Research Group, School of Dentistry, University of Lisbon, Lisbon, Portugal

**Keywords:** Cephalometrics, photographs, incisor inclination, perception, smile, aesthetics

## Abstract

**Objective:**

To correlate the clinical perception of maxillary incisor inclination from photographs of the smiling face with cephalometric measurements, using conventional incisor axis reference points and crown reference points.

**Design:**

Cross-sectional study.

**Setting:**

Department of Orthodontics, School of Dentistry, University of Lisbon (Portugal).

**Participants:**

Eight orthodontists.

**Methods:**

The perception of maxillary incisor inclination of 47 female patients (mean age 23.4 ± 1.5 years) was evaluated by eight orthodontists. The participants’ photographs (smiling frontal, smiling three-quarter and smiling profile) were shown to each assessor and a continuous visual analogue rating scale was used to assess the perception of maxillary incisor inclination. Pearson’s correlation and linear regression were calculated between each cephalometric measurement and the perception of incisor inclination.

**Results:**

Anatomical crown inclination measurements U1ac-FH (r = 0.854; *P* < 0.01) and U1ac-SN (r = 0.845; *P* < 0.01) had the highest correlation values with the assessors’ perception of maxillary incisor inclination. Conventional incisor axis measurements showed the lowest correlation values (r = 0.668–0.756).

**Conclusion:**

Cephalometric measurements of the labial surface of the anatomical crown of the maxillary incisors showed the strongest correlations with the clinical perception of maxillary incisor inclination from photographs. For optimal aesthetics, the inclination of the labial surface of maxillary incisor crown should be evaluated.

## Introduction

The smile is one of the elements for assessing the attractiveness of an individual’s face (Godinho et al., 2020; Havens et al., 2010) and the perceived attractiveness of a smile is influenced by many factors, including the position and inclination of the maxillary incisors (Shoukat Ali et al., 2021). It has been reported that an inclination of the maxillary incisors above normal cephalometric standards may be preferred by dentists, orthodontists and laypersons (Ghaleb et al., 2011).

There are several ways to measure incisor inclination (Ferrario et al., 2001; Richmond et al., 1998), but cephalometrics is still the standard method used by most orthodontists. Traditionally, the incisor axis has been used as a reference to assess the position of the maxillary incisors (He et al., 2020; Huang and Li, 2015; Yu et al., 2016). Nevertheless, the root axis may differ from the crown axis (Bryant et al., 1984). The angle between these two axes, crown to root angle, was named the ‘collum angle’ (Carlsson and Rönnerman, 1973).

Clinically, the perception of maxillary incisor inclination depends mainly on the labial surface of the crown, which is the only feature that can be easily appreciated by the patient (Ferrario et al., 2001; Shah et al., 2005).

The inclination of maxillary incisors can be evaluated in relation to several planes: sella-nasion (SN); Frankfort horizontal (FH); palatal plane (PP); and nasion-A point plane (NA) (He et al., 2020; Huang and Li, 2015; Yu et al., 2016). However, cephalometric analyses provide normative values and use internal osseous references that may be unreliable because of errors in identification and variability in their position between individuals (Baumrind and Frantz, 1971). Thus, it has been recommended that, for ideal aesthetics, maxillary incisor crown inclination should be evaluated, clinically and cephalometrically, with the patient in natural head position (NHP) (Naini et al., 2019). In addition, good facial harmony exists within a wide range of cephalometric values (Cox and van der Linden, 1971) and an orthodontic treatment that adheres strictly to a cephalometric standard does not necessarily meet aesthetic principles.

As a result, clinical evaluation of the patient is essential for diagnosis and treatment planning, and the relationship between the clinical perception of maxillary incisor inclination in a NHP and dental cephalometric measurements is important to clarify (He et al., 2020). For that reason, several studies have explored the relationship between cephalometric measurements and facial aesthetics based on smiling face photographs (He et al., 2020; Huang and Li, 2015; Yu et al., 2016). However, none of these studies specifically addresses the influence of incisor axis and crown inclination on the clinical perception of maxillary incisor inclination.

The aim of the present study was to use conventional incisor axis landmark points and crown landmark points to investigate the perception of maxillary incisor inclination in photographs of the smiling face and its relationship with cephalometric measurements of maxillary incisor inclination, and to determine the range of cephalometric values that correspond to the perception of normal inclination of the maxillary incisors.

## Methods

### Participants and assessors

The study included pre-treatment patients seeking orthodontic treatment at the Department of Orthodontics, University of Lisbon in Portugal. The inclusion criteria involved young adult female patients of European ancestry, with the maxillary incisors and canines aligned and of normal shape and size. The sample consisted only of adults to remove confounding factors, such as growth-related changes, and also because gender differences were found in previous studies (Meyer et al., 2014; Olsen and Inglehart, 2011). Patients with cavities or fillings in the anterior maxillary teeth, gingivitis or periodontal disease evident when smiling, congenital missing teeth, severe facial asymmetry and craniofacial anomalies were excluded. Data were collected over 2 months after approval by the University of Lisbon School of Dentistry Ethics Review Board. The number of participants needed for the study was calculated from data obtained in the pilot investigation to achieve a median effect size of 0.5, with α set at 0.01 and a power of 0.9.

The assessors included orthodontists, professors from the Department of Orthodontics, University of Lisbon or specialists in orthodontics with at least 5 years of clinical practice experience, who were asked to grade each participant’s maxillary incisor inclination. Eight assessors were found to be the minimal number leading to strong values of intraclass correlation (ICC ⩾ 0.8) (Godinho et al., 2020).

### Evaluation of the perception of maxillary incisor inclination

**Figure 1.**
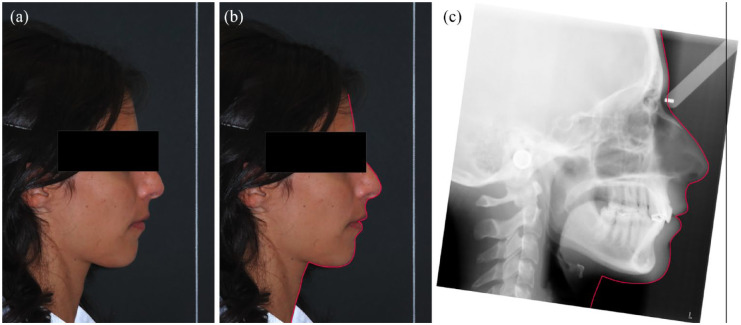
(a) Profile photograph in NHP with TV line, (b) soft tissue profile traced on the profile photograph and (c) cephalometric radiograph rotated accordingly to NHP. NHP, natural head position; TV, true vertical.

Those patients agreeing to participate in the study signed an informed consent. Four extra-oral photographs (smiling frontal, smiling three-quarter, smiling profile and profile at rest) were taken against a dark background. The face was free of makeup, glasses and jewellery. The photographs were taken in a NHP. Participants were standing, relaxed and looking straight ahead. In cases where the participant’s head was turned significantly up or down, the clinician verbally asked the participant to tilt their head forward and backward with decreasing amplitude (Tian et al., 2015) until reaching the correct orientation. A plumb line, with a suspended weight, was used to define the true vertical (TV), which was later transferred from the profile photographs at rest to the cephalometric radiographs (Figure 1).

The camera was at a standard distance of 1.5 m and at the same height as the participant’s head. The photographic equipment used was a 105-mm digital single-lens reflex camera (D80; Nikon, Tokyo, Japan) and a macro ring flash (EM-140 DG; Sigma, Aizu, Japan), with the shutter speed set at 1/125 s and the opening of the diaphragm at f10.

**Figure 2.**
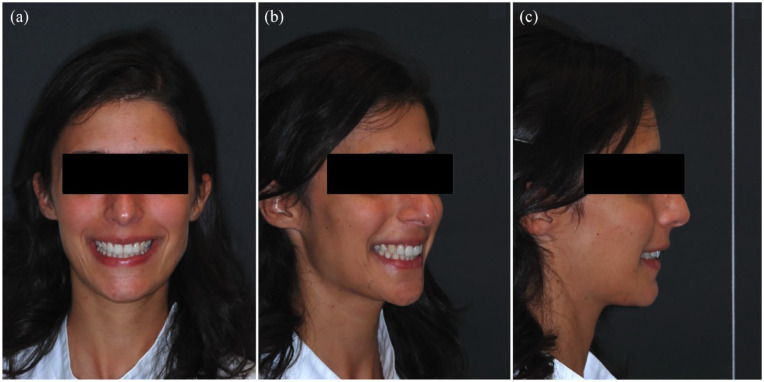
Extra-oral photographs shown to each assessor: (a) smiling frontal photograph; (b) smiling three-quarter photograph; and (c) smiling profile photograph.

**Figure 3.**
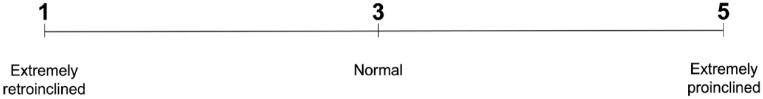
Visual analogue scale (VAS) used for assessing maxillary incisor inclination.

The three smiling photographs (Figure 2) were set up in a PowerPoint presentation (Microsoft Corp., Redmond, WA, USA), maintaining their relative size and proportion and using a dark background. These photographs were simultaneously shown to each assessor and presented in the same order. A continuous visual analogue scale (VAS) ranging from 1 (extremely retroinclined) to 5 (extremely proinclined) (Figure 3) was used to assess the overall clinical perception of maxillary incisor inclination (PMII).

Initially, assessors were only given specific instructions on using the VAS (Figure 3), and the images were not shown to them. The assessors viewed all smiling photographs first and then began the rating. They were asked not to return to any previously rated participants as they progressed through the presentation. During the rating process, each assessor was seated alone in a quiet area with reduced ambient lighting and with no time restrictions to complete the process. The macro-enabled PowerPoint presentation was always shown on the same 16-inch computer screen. Means and standard deviations (SD) of the scores by the assessors were calculated for each participant’s set of photographs.

### Cephalometric measurement of maxillary incisor inclination

**Figure 4.**
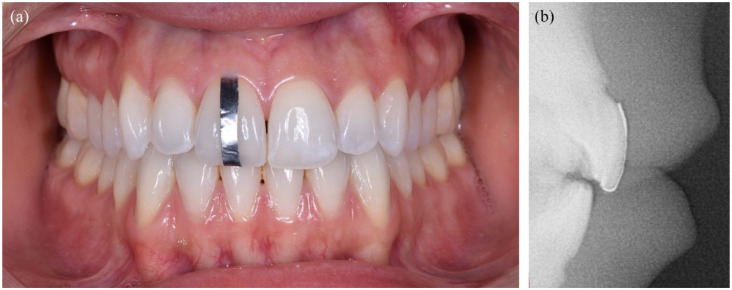
(a) Metal strip applied before taking the cephalometric radiographs and (b) radiographic image.

A metallic strip was placed on the midline of the labial surface of the most protruded maxillary incisor (Figure 4a), from the maxillary limit of the gingival margin (gm point) to the midpoint of the incisal edge (Ui point), allowing an easy identification of the gingival margin and of the incisal edge on the cephalometric radiographs (Figure 4b).

**Figure 5.**
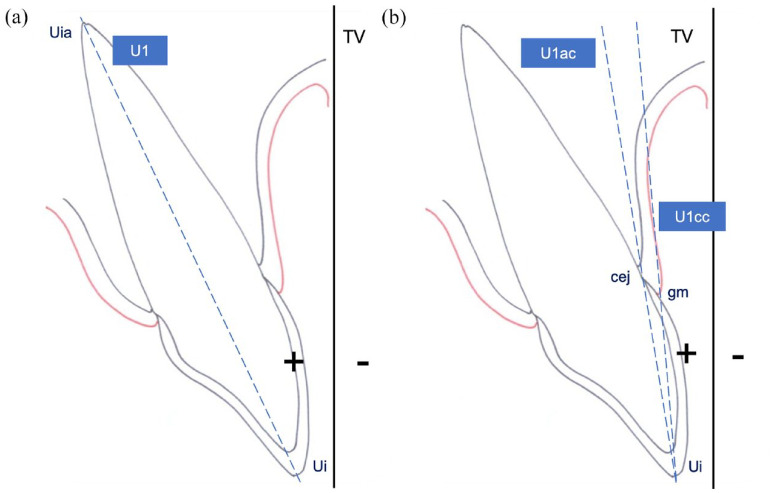
(a) Conventional and (b) crown landmark points for maxillary incisor inclination measurement.

The pre-treatment cephalometric radiographs were taken using a cephalostat, and each participant’s radiographs and photographs were imported into the NemoCeph cephalometric software (Nemotec, Madrid, Spain). The radiographs were oriented by transferring the TV from the photograph to the film (Figure 1). To measure maxillary incisor inclination, three different lines were used: one corresponding to the tooth long axis (U1: conventional cephalometric measurement), and the other two related to the crown labial surface (U1cc: clinical crown and U1ac: anatomical crown measurements) (Table 1 and Figure 5). Maxillary incisor inclination was determined according to five reference planes: TV; NA; SN; FH; and PP. The same investigator traced all 47 lateral radiographs. To determine the error of the cephalometric method, 15 randomly selected cephalograms were retraced by the same operator 1 month later.

**Table 1.** Landmark points for measuring maxillary incisor inclination.

Conventional landmark points
U1 – line segment from the maxillary incisor apex (Uia) to the midpoint of the incisal edge of the most protruded maxillary incisor (Ui)
Crown landmark points
U1ac – line segment from the midpoint of the cementoenamel junction (cej) to the midpoint of the incisal edge of the most protruded maxillary incisor (Ui) U1cc – line segment from the upper limit of the contour of the gingival margin (gm) to the midpoint of the incisal edge of the most protruded maxillary incisor (Ui)

### Statistical analysis

The statistical analysis was performed using SPSS version 27.0 for Macintosh (IBM Corp., Armonk, NY. USA). The Shapiro–Wilk test was used to verify the normality of the distribution of cephalometric measurements and rating scores. The mean, standard deviation and 95% confidence interval were calculated for each cephalometric measurement. The reliability of the PMII scores was evaluated both in a pilot investigation and in the main study, by having the PMII scored by the assessors a second time, 1 month later. The ICC was used to assess the random errors of the cephalometric method and of the scores of incisor inclination perception.

Pearson’s correlation was used, followed by a linear regression to assess the relationship between each cephalometric measurement and the PMII as evaluated by the rating scores. Due to multiple testing the level of significance was set at 0.01. The average between the first and second assessors’ scores was used for Pearson’s correlation and linear regression assessment.

## Results

The final sample comprised 47 female participants (mean age = 23.4 ± 1.5 years; age range = 21–28 years). The consistency between the first and the second scores of the photographs was excellent (ICC = 0.99). All ICCs for the repeated cephalometric measurements were greater than 0.93, indicating excellent intra-operator reliability.

Descriptive statistics and the results of the Shapiro–Wilk test for the cephalometric measurements of maxillary incisor inclination are presented in Tables 2 and 3. Normal distribution was verified for each cephalometric measurement.

**Table 2.** Skeletal cephalometric measurements of the sample (n=47).

Measurements (°)	Mean ± SD
SNA	80.1 ± 3.1
SNB	76.7 ± 3.2
ANB	3.4 ± 2.2
SN-PP	8.5 ± 3.4
SN-MP	35.9 ± 5.4

SD, standard deviation.

**Table 3.** Descriptive statistics and Shapiro–Wilk test results for the dental cephalometric measurements (n=47).

Measurements (°)	Mean ± SD	Range	*P* value*
U1-TV	19.0 ± 6.6	1.9–30.0	0.326
U1-NA	20.2 ± 7.0	7.4–33.3	0.216
U1-SN	100.3 ± 7.3	86.9–116.5	0.226
U1-FH	110.9 ± 7.6	95.5–127.6	0.709
U1-PP	108.8 ± 7.2	97.1–122.2	0.025
U1ac-TV	1.7 ± 7.1	−17.7–13.5	0.362
U1ac-NA	3.0 ± 7.6	−10.3–17.8	0.288
U1ac-SN	83.1 ± 7.7	68.6–101.0	0.633
U1ac-FH	93.7 ± 8.2	75.9–112.1	0.983
U1ac-PP	91.6 ± 7.6	78.3–104.5	0.055
U1cc-TV	−2.9 ± 6.9	−23.1–9.3	0.646
U1cc-NA	−1.7 ± 7.5	−16.0–12.2	0.419
U1cc-SN	78.4 ± 7.4	63.4–95.6	0.871
U1cc-FH	89.0 ± 8.0	70.5–103.4	0.466
U1cc-PP	86.9 ± 7.3	72.9–100.2	0.252
U1cc-U1	21.9 ± 3.9	11.4–29.0	0.394
U1ac-U1	17.2 ± 3.3	10.5–23.8	0.655

* Using the Shapiro–Wilk test to verify normality in sample distribution.

SD, standard deviation.

Table 4 shows the results of Pearson's correlation and linear regression between maxillary incisor inclination scores and dental cephalometric measurements. All 15 cephalometric measurements correlated significantly with the PMII scores of smiling face photographs with *P* values <0.01. The absolute values of the correlation coefficients were in the range of 0.668–0.854. The anatomical crown inclination measurements U1ac-FH (*r* = 0.854; *P* < 0.01) and U1ac-SN (*r* = 0.845; *P* < 0.01) obtained the highest correlation values, demonstrating an extremely high correlation with the assessors’ perception of incisor inclination. Conventional measurements of maxillary incisor axis inclination showed the lowest correlation values. Regarding the reference planes, the cephalometric measurements related to the palatal plane showed the lowest correlation values with perception of incisor inclination obtained from a clinical evaluation.

**Table 4.** Pearson correlation and linear regression between the average score of maxillary incisor inclination given by the assessors and the cephalometric measurements (n=47).

Measurements	Pearson		Linear regression		*P* value
	r	R^2^	Intercept	Slope	
U1-TV	0.709*	0.502	1.569	0.069	<0.001
U1-NA	0.721*	0.520	1.557	0.066	<0.001
U1-SN	0.705*	0.497	−3.281	0.061	<0.001
U1-FH	0.756*	0.571	−4.224	0.064	<0.001
U1-PP	0.668*	0.447	−3.588	0.059	<0.001
U1ac-TV	0.833*	0.693	2.754	0.075	<0.001
U1ac-NA	0.835*	0.697	2.677	0.070	<0.001
U1ac-SN	0.845*	0.715	−2.996	0.071	<0.001
U1ac-FH	0.854*	0.729	−3.361	0.067	<0.001
U1ac-PP	0.805*	0.648	−3.351	0.068	<0.001
U1cc-TV	0.805*	0.648	3.102	0.075	<0.001
U1cc-NA	0.798*	0.637	3.001	0.069	<0.001
U1cc-SN	0.816*	0.665	−2.628	0.070	<0.001
U1cc-FH	0.828*	0.685	−3.041	0.067	<0.001
U1cc-PP	0.775*	0.601	−2.995	0.068	<0.001

* The correlation is significant (*P* < 0.01).

**Figure 6.**
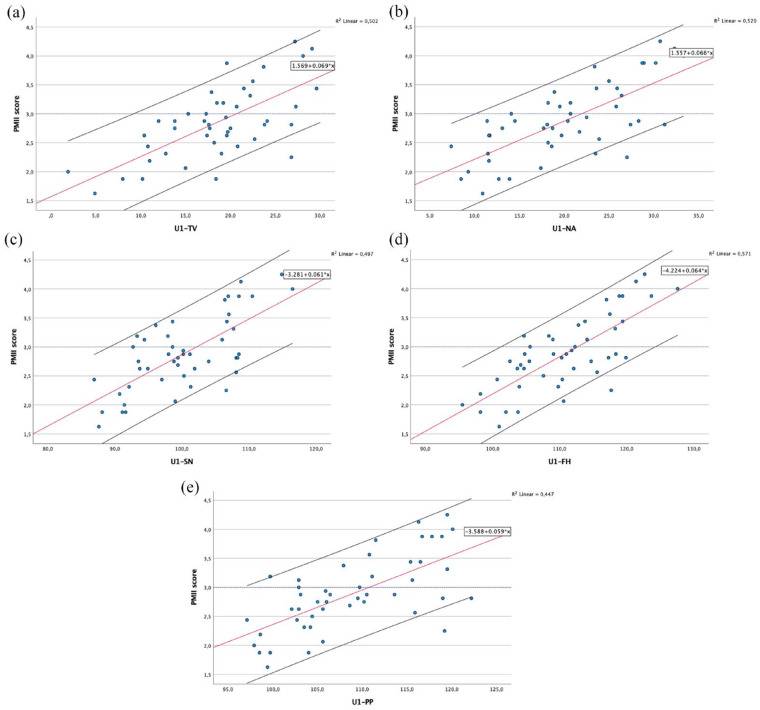
Scatterplots and fitted regression line of data for maxillary incisor conventional measurements (U1) in relation to: (a) true-vertical line; (b) NA line; (c) sella-nasion line; (d) Frankfurt horizontal plane; and (e) palatal plane.

**Figure 7.**
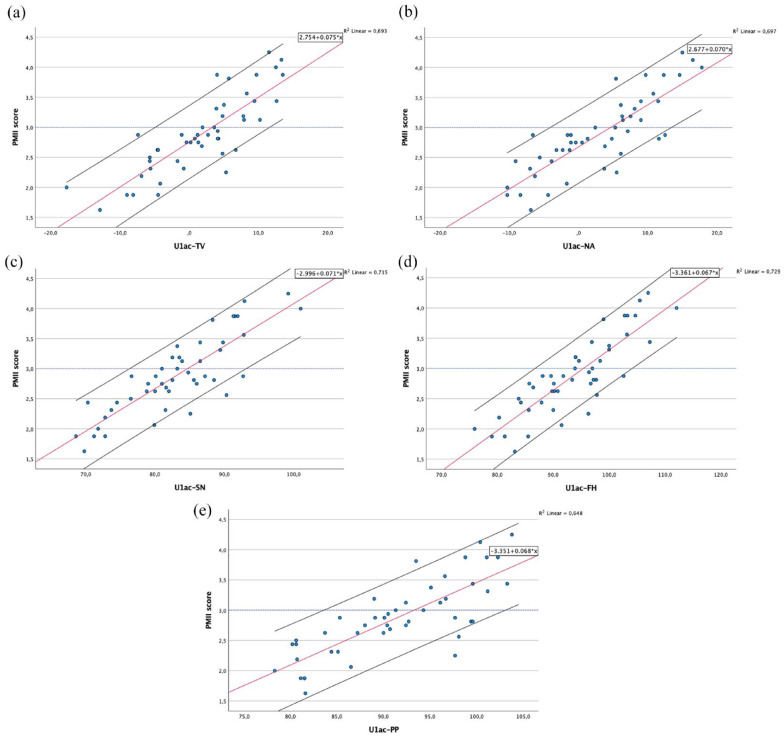
Scatterplots and fitted regression line of data for maxillary incisor anatomical crown measurements (U1ac) in relation to: (a) true-vertical line; (b) NA line; (c) sella-nasion line; (d) Frankfurt horizontal plane; and (e) palatal plane.

**Figure 8.**
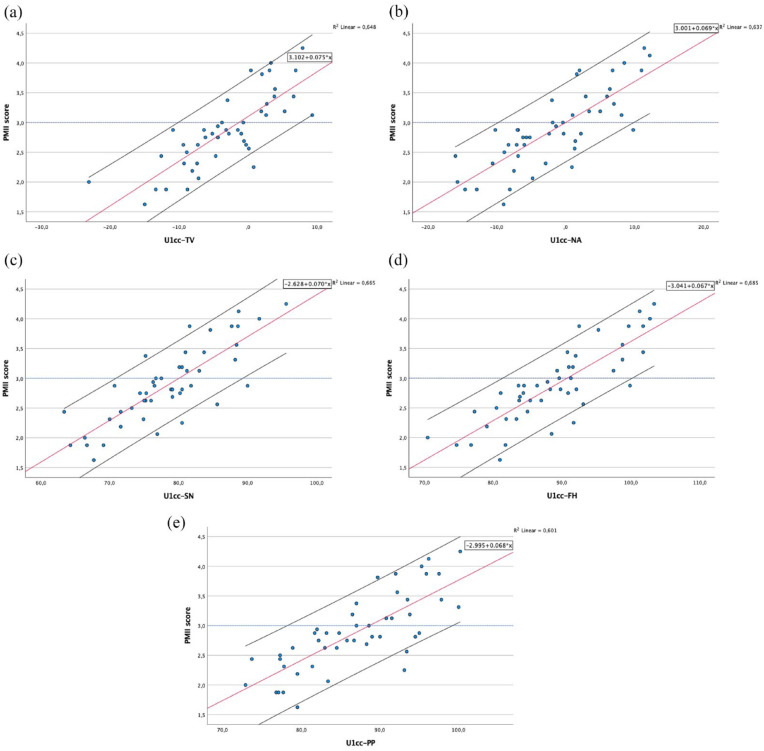
Scatterplots and fitted regression line of data for maxillary incisor clinical crown measurements (U1cc) in relation to: (a) true-vertical line; (b) NA line; (c) sella-nasion line; (d) Frankfurt horizontal plane; and (e) palatal plane.

Scatterplots for each of the cephalometric measurements are given in Figures 6–8, showing the association between the PMII scores (y-axis) and each of the cephalometric measurements (x-axis). The fitted regression line is represented by a red line, with the black lines below and above representing the 90% confidence interval (CI).

All 15 cephalometric measurements (CM) showed a strong linear relationship making it possible to establish the regression equations (PMII score = slope × CM + intercept) between each of the 15 cephalometric measurements and the PMII score of facial photographs listed in Table 3. In order to estimate the cephalometric values and the confidence intervals corresponding to the PMII score 3 (Table 5), the correlation was made the other way round (CM = slope × PMII score + intercept).

**Table 5.** Cephalometric measurements estimated for normally inclined maxillary incisors (score 3) through the linear regression line equation.

Measurement	Estimated value	90% CI
U1-TV	19.8	11.9–27.8
U1-NA	21.1	12.7–29.5
U1-SN	101.2	92.3–110.2
U1-FH	111.9	103.4–120.4
U1-PP	109.7	100.5–118.9
U1ac-TV	2.8	−4.0–9.6
U1ac-NA	4.1	−3.1–11.3
U1ac-SN	84.2	77.2–91.2
U1ac-FH	94.9	87.6–102.3
U1ac-PP	92.7	85.0–100.4
U1cc-TV	−1.9	−8.9–5.1
U1cc-NA	−0.6	−8.3–7.1
U1cc-SN	79.5	72.1–86.9
U1cc-FH	90.2	82.5–97.9
U1cc-PP	88.0	80.0–95.9

CI, confidence interval.

## Discussion

### Summary

The maxillary incisors have a great influence on a patient’s smile (Shoukat Ali et al., 2021), in such a way that is extremely important to consider the clinical perception of the inclination of the maxillary incisors.

In the present study, only patients with aligned maxillary anterior teeth were included. This was done to have similar crown inclinations among all the anterior teeth and reduce doubt during the rating process. In addition, patients with cavities, fillings or periodontal problems evident when smiling, which may influence the attractiveness of the smile, were excluded so there was no bias in the perception of incisor inclination when the photographs were assessed.

In any study involving head positioning and craniofacial analysis, efforts should be made to standardise the head positioning technique and reduce errors in cephalometric measurements. In the present study, the NHP was established without the use of an external reference, such as a mirror, avoiding the disadvantage that the position obtained may not be the one usually used by the participant outside the experimental situation (Solow and Tallgren, 1971). All cephalometric radiographs were taken on the same cephalostat, with the TV transferred from the photograph to the film. This added one more step to the process and may have introduced errors in the method. However, it has been shown that the transfer of a vertical line from photographs to radiographs is a clinically acceptable and valid method (Bister, 2002; Dvortsin et al., 2011; Zebeib and Naini, 2014). It has been observed clinically that because of the cephalostat ear rods, patients tend to hold their head in an unnaturally extended or flexed position when radiographs are taken, which may give misleading outcomes (Dvortsin et al., 2011; Yoon et al., 2001). On the contrary, when photographs are taken, patients appear to be more relaxed (Dvortsin et al., 2011) and a reproducible NHP can be obtained in both pitch and roll head orientation (Tian et al., 2015; Weber et al., 2013). Therefore, reorienting radiographs according to standardised photographs is a good and simple method (Dvortsin et al., 2011).

In the present study, we found a large cephalometric range of maxillary incisor inclination that can be perceived as normal for all measurements. This can be explained, in part, by the fact that we used participants’ unaltered colour photographs and not digital modifications of the same photograph, as in other studies (Cao et al., 2011; Doshi et al., 2017; Ghaleb et al., 2011; Naini et al., 2019; Shoukat Ali et al., 2021). Unaltered colour photographs, through the addition of facial distractions such as skin and hair colour, give a more realistic representation of perceived clinical features (Maple et al., 2005) and more accurately reflect individual variability and the clinical environment of orthodontic practice.

The maxillary incisors should be considered a part of the face, both in the frontal and lateral perspectives (Doshi et al., 2017). Olsen and Inglehart (Olsen and Inglehart, 2011) reported that the three-quarter photograph may be interpreted as more representative in case of social interaction with other people than a lateral view. For these reasons, in our study, assessors scored the smiling photographs in the frontal, three-quarter and profile views. Full-face photographs were used rather than smile close-ups to allow for a thorough evaluation of the patient and to reflect routine orthodontic clinical practice.

### Comparisons with other studies

Some studies have explored the correlation between cephalometric measurements and facial aesthetics assessed in photographs (He et al., 2020; Huang and Li, 2015; Yu et al., 2016). However, these studies measured maxillary incisor inclination using only the incisor axis as reference. In this study, the clinical perception of incisor inclination was correlated with cephalometric measurements using conventional landmark points as well as landmarks on the labial surface of the crown.

In the present sample, measurements of the U1 axis showed the lowest correlation values and this could be explained because the root long axis and the crown long axis often do not coincide (Bryant et al., 1984). Therefore, drawing a line between the incisal edge and the apex of the incisor may not reflect the crown inclination in participants with diverse crown-root angles (Richmond et al., 1998). The PMII depends mainly on the labial surface of the crown and is only partially reflected by the axis of the crown, and even less by the long axis of the incisors. In fact, during orthodontic treatment, maxillary incisors perceived as having a normal inclination may develop an improper root position.

A previous study (Naini et al., 2019) measured maxillary incisor inclination by using a line tangent to the labial surface. However, cartoon-type two-dimensional manipulated images were used, not radiographs. In real patients, as the gingival margin is not visible on radiographs, a tangent to the centre of the clinical crown cannot be accurately determined on cephalometric radiographs. Furthermore, due to the variation in curvature of the labial surface of the maxillary incisor (van Loenen et al., 2005) from the incisal edge to the cemento-enamel junction, lines tangent to the labial surface may not represent the general inclination of the labial surface of the maxillary incisor.

### Implications for clinical practice

In this study, the U1ac-FH (*r* = 0.854; *P* < 0.01) and U1ac-SN (*r* = 0.845; *P* < 0.01) measurements obtained the highest correlation values, demonstrating an extremely high correlation with the clinical perception of the inclination of the maxillary incisors. It could be expected that the measurements related to U1cc would have higher correlation values than those related to U1ac, since the former are related to the clinical crown and the latter to the anatomic crown. However, correlations related to U1cc measurements were slightly lower, possibly because positioning and stabilising the metal strip in the mouth is a difficult process. Therefore, as the difference in correlation values between the two sets of measurements is small, U1ac measurements may be valuable in daily clinical practice, due to its ease of use and good repeatability.

Cephalometric measurements related to the palatal plane showed the lowest correlation values, demonstrating low correlation with perception of incisor inclination. This may be due to variations in the inclination of the palatal plane between participants and underscores the need to consider other clinical and cephalometric variables when planning the treatment of orthodontic patients.

Because taking cephalometric radiographs in NHP provides a physiologically natural position of the head, one could expect that the cephalometric measurements related to TV would show higher correlation values than those related to FH. Several studies in the literature have concluded that the clinically significant variability in the inclination of SN and FH in relation to the true horizontal plane makes their use unreliable (Lundström et al., 1995; Moorrees, 1994; Zebeib and Naini, 2014). However, unlike these studies, our study demonstrated similar correlation values for SN and FH for both conventional measurements and measurements of the labial surface of the crown, possibly because the mean inclination of FH to TV is approximately 90° and close to the true horizontal. Solow and Tallgren (1971) and Zebeb and Naini (2014) mentioned the same in their studies.

It should also be emphasised that a regression equation can be established between each of the 15 cephalometric measurements and the PMII score of the smiling face photographs, which makes it possible to estimate the value of PMII score from a given cephalometric value. Likewise, it is possible to make the estimation the other way round and find the cephalometric value corresponding to a given PMII score. The results of this investigation seem to indicate that with the patient in a NHP, the inclination of the labial surface of the maxillary incisor crown perceived as normal will be 2.8° in relation to the TV, and approximately perpendicular to the FH (U1ac-FH = 94.9°), as shown in Table 5.

Our study highlights the importance of patient clinical evaluation and some of the limitations of conventional cephalometric measurements regarding the perception of maxillary incisor inclination. In fact, cephalometric standards should not be the main goal of orthodontics, but only a general guide to support our clinical decision, giving greater importance to the physical evaluation.

### Limitations and implications for research

As with any study, the limitations of the sample must be considered when generalising the study results. This study was performed using unaltered extra-oral photographs of female patients. Facial features, such as skin colour, tone and hairstyle, may have influenced the PMII. The assessors’ panel was composed only of orthodontists and it is known that the opinions of professionals sometimes do not coincide with the perceptions and expectations of laypersons (Kokich et al., 2006; Meyer et al., 2014). Thus, it would be of interest to assess the PMII by laypersons.

The sample consisted of only young women. Further investigations with men could also provide additional conclusions regarding the contribution of incisor inclination to smile attractiveness.

## Conclusions

All 15 dental cephalometric measurements were strongly correlated with the scores evaluating the perception of maxillary incisor inclination in facial photographs. Cephalometric measurements of the inclination of the labial surface of the maxillary incisor crown showed stronger correlations with the perception of maxillary incisor inclination than did incisor axis measurements. For optimal aesthetics, the inclination of the labial surface of the maxillary incisor crown should be evaluated. With the patient in a NHP, in this study, the inclination of the maxillary incisor anatomical crown was perceived as normal when it is approximately parallel to the TV (U1ac-TV = 2.8°) and perpendicular to the FH (U1ac-FH = 94.9°).
